# Diffuse myocardial fibrosis in patients after Fontan operation: a T1 relaxometry magnetic resonance pilot study

**DOI:** 10.1186/1532-429X-18-S1-O28

**Published:** 2016-01-27

**Authors:** Atsuko Kato, Eugenie Riesenkampff, Deane Yim, Shi-Joon Yoo, Mike Seed, Lars Grosse-Wortmann

**Affiliations:** grid.42327.300000000404739646Cardiology, The Hospital For Sick Children, Toronto, ON Canada

## Background

Patients with single ventricle (SV) circulations are at risk for heart failure, frequently burdened by both systolic and diastolic cardiac dysfunction. The mechanisms of adverse remodeling of functionally univentricular hearts are incompletely understood. Diffuse myocardial fibrosis is one of the candidate morphological substrates, for which cardiac magnetic resonance (CMR) derived native T1 times and extracellular volume fraction (ECV) have been used as markers. The purpose of this pilot study was to explore whether there is evidence of accelerated myocardial fibrosis in young children after the Fontan operation using CMR.

## Methods

Consecutive pediatric SV patients after the Fontan operation who were referred for a clinical CMR examination underwent T1 mapping using a modified look-locker inversion recovery approach. Patients with two relatively balanced ventricles were excluded. Native T1 times and ECV in the free wall of the dominant ventricle were measured along with ventricular volumes and mass. Results were compared with those from healthy pediatric controls as well as between dominant left (LVs) and right ventricles (RVs).

## Results

Thirteen SV patients (7 with a dominant LV and 6 with a dominant RV), aged 8.8 ± 4.9 years and 28 healthy control children (13.8 ± 2.5 years; p = 0.003) were included. Despite the younger age native T1 times were longer in the dominant ventricles of SV patients than those in the LVs of the control group (1013 ± 26 ms in dominant LVs [p = 0.007], and 1056 ± 36 ms in dominant RVs [p = 0.01] vs. 982 ± 26 ms in LVs of controls, Figure). ECV in SV patients with dominant LVs was similar to that of controls while ECV in dominant RVs was increased (23 ± 3% in dominant LVs [p = 0.5], and 29 ± 3% in dominant RVs [p = 0.002], vs. 22 ± 3% in LVs of controls, Figure). Among univentricular hearts, there was a trend towards shorter native T1 times in dominant LVs (p = 0.07). ECV was significantly lower in LVs than those in dominant RVs (p = 0.01). Indexed ventricular volumes and mass, ejection fraction, cumulative cardiopulmonary bypass or cross-clamp times did not correlate with native T1 or ECV. Native T1 times positively correlated with the age at the bidirectional cavopulmonary connection (r^2^ = 0.71, p = 0.001).

## Conclusions

Pre- and post contrast T1 measurements are feasible in children with functionally SVs. Higher T1 times suggest an increased likelihood of myocardial fibrosis in Fontan patients, especially in patients with a dominant RV. Whether the known greater risk for long-term complications following the Fontan completion in non-dominant LVs is a function of their increased fibrosis burden remains to be investigated.

**Figure 1:**
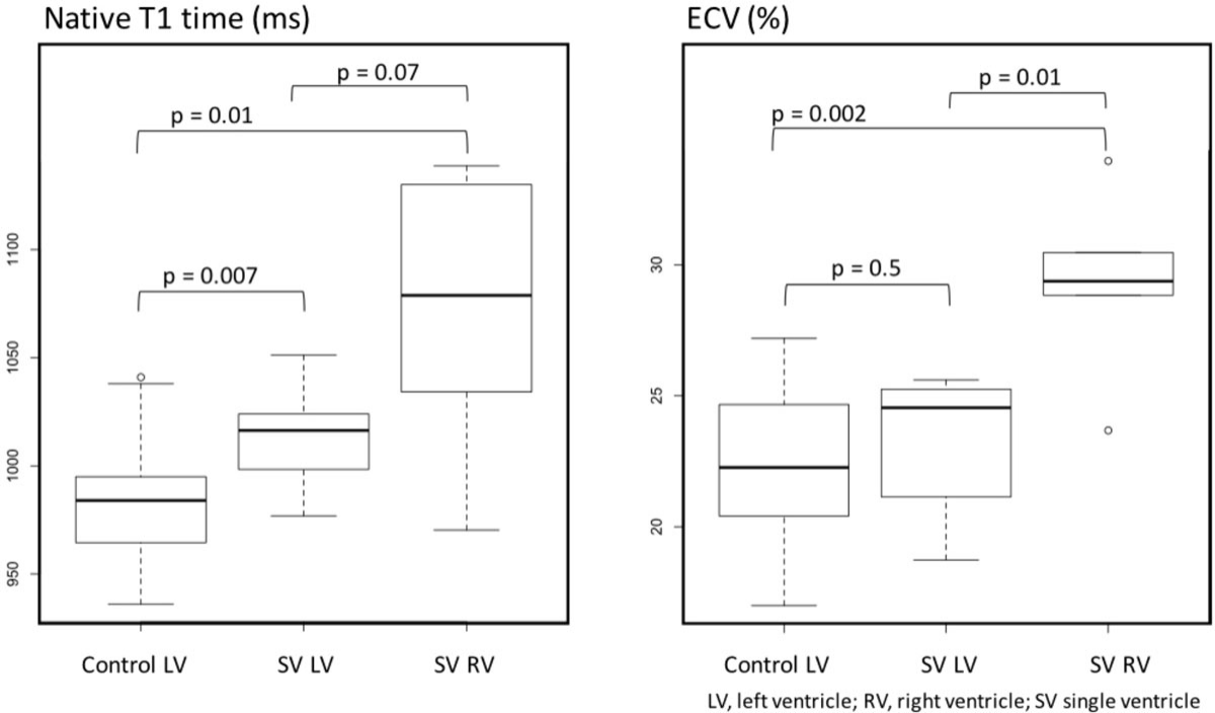
**Student's T-test comparison of T1 parameters between the dominant ventricle in SV group and Control group**.

